# Impedance spectroscopy for *in situ* and real-time observations of the effects of hydrogen on nitrile butadiene rubber polymer under high pressure

**DOI:** 10.1038/s41598-019-49692-y

**Published:** 2019-09-10

**Authors:** Jae Kap Jung, Sang Koo Jeon, Kyu-Tae Kim, Chang Hoon Lee, Un Bong Baek, Ki Soo Chung

**Affiliations:** 1 Center for Materials and Energy Measurement, Korea Research Institute of Standards and Science, Daejeon, 34113 Korea; 2 Department for Safety Engineering, Pukyong National University, Busan, 48513 Korea; 3 Center for Electromagnetic Metrology, Korea Research Institute of Standards and Science, Daejeon, 34113 Korea; 4 Department of Biochemical and Polymer Engineering, Chosun University, Gwangju, 61452 Korea; 5 Department of Physics, and The Research Institute of Natural Science, Gyeongsang National University, Jinju, 52828 Korea

**Keywords:** Characterization and analytical techniques, Characterization and analytical techniques

## Abstract

Nondestructive impedance spectroscopy (IS) was developed and demonstrated to detect the effects of hydrogen on nitrile butadiene rubber exposed to hydrogen gas (H_2_) at high pressures up to 10 MPa. IS was applied to obtain an *in situ* and real-time quantification of H_2_ penetration into and its desorption out of rubber under high pressure. The diffusion coefficients of H_2_ were also obtained from the time evolution of the capacitance, which were compared with those obtained by thermal desorption gas analysis. The *in situ* measurements of the capacitance and the dissipation factor under various pressures during cyclic stepwise pressurization and decompression demonstrated the diffusion behaviour of H_2_, the phase of the rubber under high pressure, the transport properties of H_2_ gas, and the physicochemical interaction between H_2_ and the rubber. These phenomena were supported by a COMSOL simulation based on the electric current conservation equation and scanning electron microscopy (SEM) observations.

## Introduction

Hydrogen gas (H_2_) is expected to be a future clean-energy source to mitigate global warming and the exhaustion of fossil fuels. The use of H_2_ as an energy carrier requires safe materials to be used in the H_2_ infrastructure^1–3^. To satisfy this safety requirement, the embrittling effects of H_2_ exposure have been studied in steel, stainless steel, aluminium, and alloys^4–8^.

However, many kinds of rubber polymers are used as sealants in H_2_ environments: polytetrafluoroethylene (PTFE) is used as a sealant in mechanical compressors, and nitrile butadiene rubber (NBR), ethylene propylene diene monomer (EPDM), and fluoroelastomer (FKM) rubbers are used as sealants and gaskets in valves and pipelines^9^. Research on rubber polymers used for gas sealants and liners at the H_2_ station has focused on understanding the origin of explosive failure by decompression and the swelling behaviour of O-ring rubber under high-pressure H_2_ ^10–12^. Research on how H_2_ affects the physical properties and morphology after pressurization and decompression has been conducted, but *in situ* measurements during pressurization and decomposition under high pressure have rarely been performed.

In this study, by using impedance spectroscopy (IS) to measure the capacitance and the dissipation factor (DF), we performed *in situ*, real-time monitoring of the dynamic macroscopic behaviour of the penetration of the H_2_ molecule into O-ring rubber and its desorption from the rubber during the processes of pressurization and decompression under high-pressure H_2_ gas. The results of the impedance measurement for NBR at various pressures and exposure times to H_2_ gas were analysed. The quantities considered include H_2_ diffusion, interfacial polarization, plasticization, permeation properties, and the physicochemical interaction between H_2_ and the polymer during the processes of pressurization and decompression. The results of scanning electron microscopy (SEM) and a COMSOL simulation support the IS observations.

## Experimental

### Sample preparation

**Figure 1:**
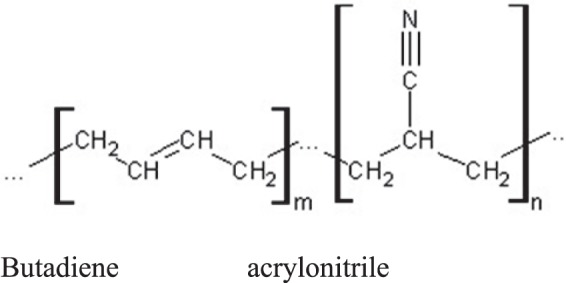
Molecular structure of the NBR copolymer of acrylonitrile and butadiene; m: number of butadiene moieties; n: number of acrylonitrile moieties.

NBR (Fig. 1, Table 1) is a synthetic rubber copolymer of acrylonitrile and butadiene. NBR is widely used as a sealing material due to its excellent gas resistance. The NBR used in this study was synthesized by a Korean domestic company, and 22% carbon black was included as a filler during the fabrication of the NBR specimen.

**Table 1:** Chemical composition of the NBR specimen.

Chemical	Weight ratio (%)
NBR	72
Carbon black	22
Dicumyl peroxide	1
ZnO	2
Stearic acid	1
Dioctyl adipate	2
Total	100

### Impedance spectroscopy system

**Figure 2:**
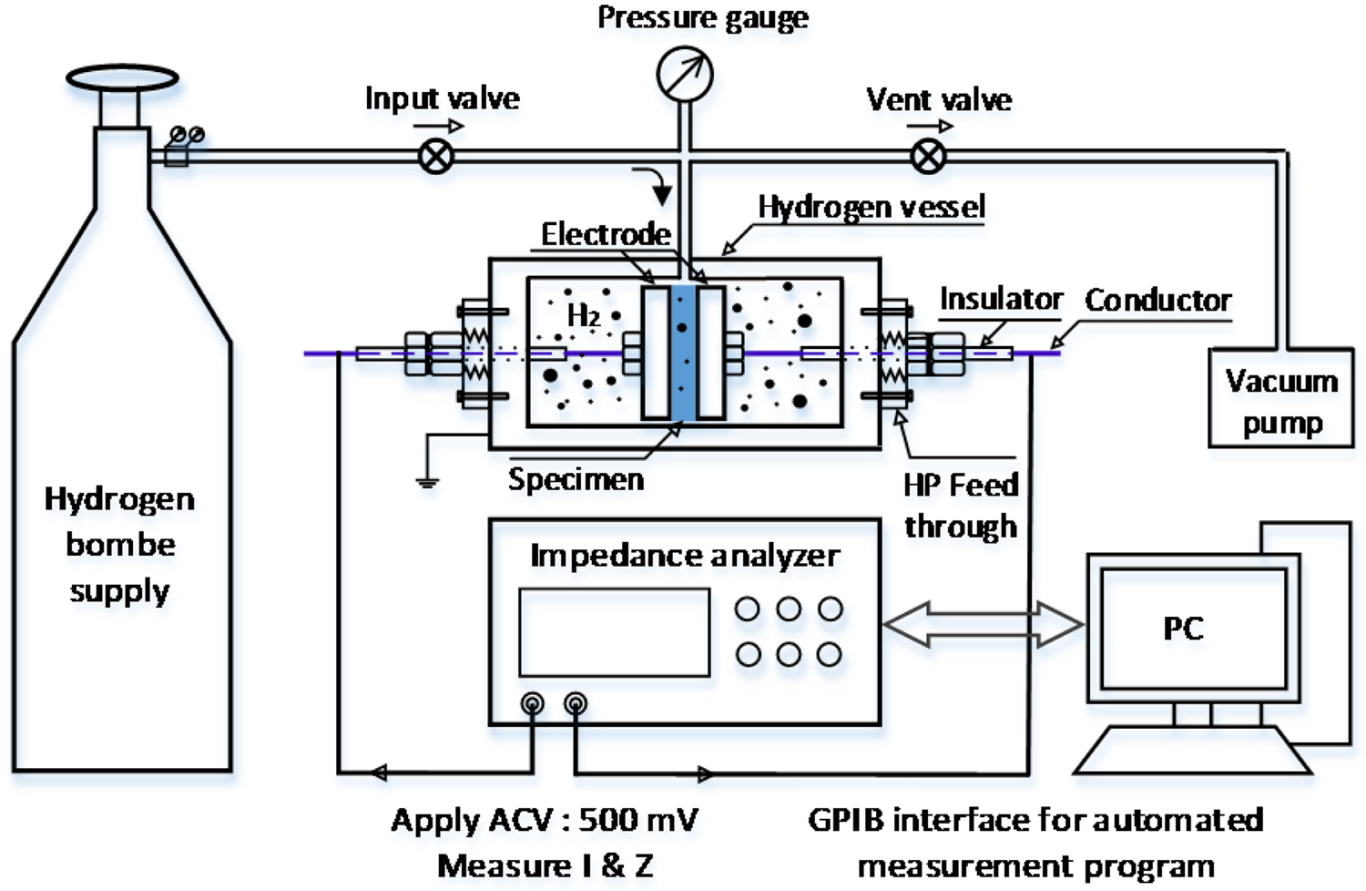
Configuration of the whole *in situ* impedance spectroscopy system under high-pressure H_2_.

The IS system (Fig. 2) for the *in situ* measurement under high pressure comprises a hydrogen gas supply, input and vent valves, a pressure gauge, a hydrogen vessel, and an impedance analyser with a general purpose interface bus (GPIB) interface to a PC. The hydrogen vessel contains the specimen, electrodes and a high-pressure feed-through to withstand the seal at high pressure. The NBR used for this measurement was disc-shaped, 49 mm in diameter and 2 mm in thickness. The specimen was inserted between two electrodes and later fixed in place by mechanical pressure. The variation in the impedance of the specimen was monitored in real time using an Agilent 4294 A impedance analyser and an automated measurement program at the frequencies of the applied voltage of 40 Hz ≤ *frequency* ≤ 10000 Hz. The *in situ* capacitance *C*_NBR_ and DF *DF*_NBR_ were simultaneously measured as a function of pressure and exposure time for several days.

### Thermal desorption analysis

Thermal desorption gas analysis (TDA) was performed using a 7890A analyser with a pulsed discharge detector from Agilent. To quantify the charged hydrogen content in the NBR, a specimen that had been exposed to H_2_ gas at 10 MPa for 24 h was mounted in the tube furnace of the analyser. The specimen was 20 mm in diameter and 2 mm in thickness. The release of H_2_ gas was measured by gas chromatography every 5 min for the first hour and then at intervals of 1 h for 16 h.

## Results and Discussion

### COMSOL simulation

**Figure 3:**
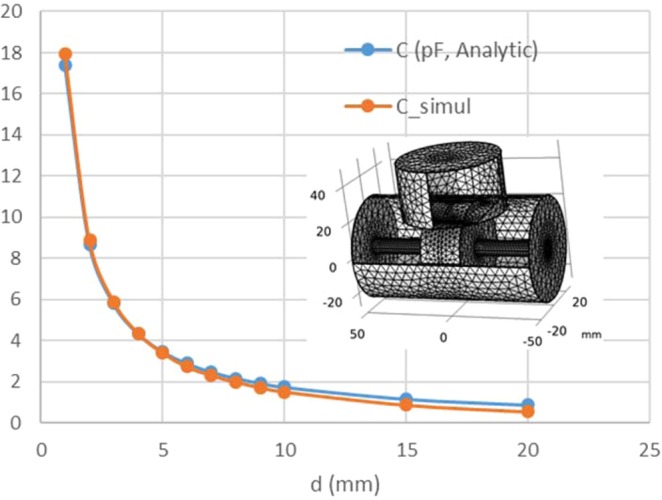
COMSOL software simulation results. Inset: the mesh structure of the modelled vessel. C_simul: COMSOL software simulation results; C_analytic: capacitance values predicted by an ideal parallel-plate model.

To quantify the relationship between the measured *C*_NBR_ and the relative dielectric constant *ε*_r_ of the rubber, COMSOL software was used to model the hydrogen vessel and the electrodes (Fig. 3). The electric current conservation equations, ***J*** = *σ****E*** + ***J***_e_, ***E*** = *−***∇***V*, where ***J*** is the current density, *σ* is the conductivity, ***J***_e_ is the external current density, ***E*** is the electric field, and *V* is the electric potential, are solved with a boundary condition set by a ground domain at one electrode extending to the outer cylinder and a 1 V potential domain at the other electrode. A default mesh condition with a fine mesh size was used. The capacitance is equal to the total charge accumulated at the electrode, because we applied a unit potential of 1 V. The charge was calculated by surface integration of the surface charge over the front and side faces of the counter electrode, including the centre rod. In order to simulate the grounded vessel, the surface charge integration was carried over the counter electrode only excluding the vessel. The COMSOL simulations (Fig. 3, C_**-**_simul) show the capacitance *C* versus the electrode gap *d*. For *d* < 1 mm, the C_**-**_simul deviated from the capacitances (*C*_**-**_analytic) calculated using an ideal parallel-plate model, *C*_NBR_ = *ε*_0_ · *R*^2^/*d* (where *R* is the radius of the parallel electrode face), because of the limits of the geometric precision in the mesh modelling. The deviation between *C*_**-**_simul and *C*_**-**_analytic for *d* > 10 mm is attributed to a stray field that primarily forms between the side faces of the electrodes. It should be noted that the stray field between electrode faces and the inner faces of the vessel does not make any effect in capacitance because the vessel is electrically grounded. This is the main reason why the deviation for *d* > 10 mm is not very large. For high sensitivity and to avoid the stray field effect, we chose *d* = 2 mm. Another simulation with the rubber inserted between the electrodes was performed by varying the relative dielectric constant of the rubber material for *d* = 2 mm. The result was a simple linear relationship: *C*_NBR_ = 0.31 + 8.22*ε*_r_ [pF]. The linear relationship is expected to be maintained for other values of *d*.

### H_2_ effect on the impedance spectra

**Figure 4:**
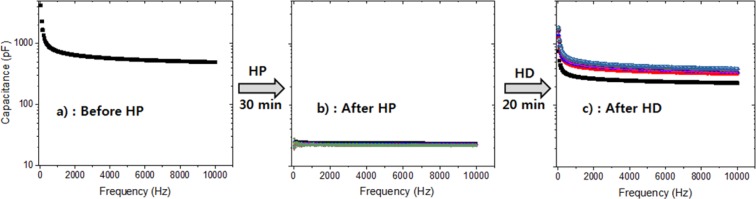
Effect of hydrogen on the capacitance spectra versus frequency for NBR: (**a**) before HP, (**b**) after HP, and (**c**) after HD. The logarithmic scale is the same in all figures.

The effect of H_2_ on the spectra of *C*_NBR_ versus *frequency* for NBR in the sequential overall processes was quantified in three steps. (1) The *C*_NBR_ spectra versus frequency were measured before H_2_ pressurization (HP) (Fig. 4a). (2) The pressure was increased to 10 MPa over 30 min, and the *C*_NBR_ spectra versus frequency were subsequently measured after HP at 10 MPa for 25 h (Fig. 4b; several lines overlay). (3) H_2_ decompression (HD) was conducted for 20 min, and then the *C*_NBR_ spectra versus frequency were measured after HD for 30 h (Fig. 4c; several lines overlay). The capacitance decreased by more than an order of magnitude after HP and then recovered to almost its original value after HD. The *DF*_NBR_ spectra versus frequency showed behaviour similar to that of the *C*_NBR_ spectra. The observed large change in both *C*_NBR_ and *DF*_NBR_ implies that IS responds very rapidly to pressurization and decompression and is therefore an appropriate tool for detecting the effects of H_2_.

**Figure 5:**
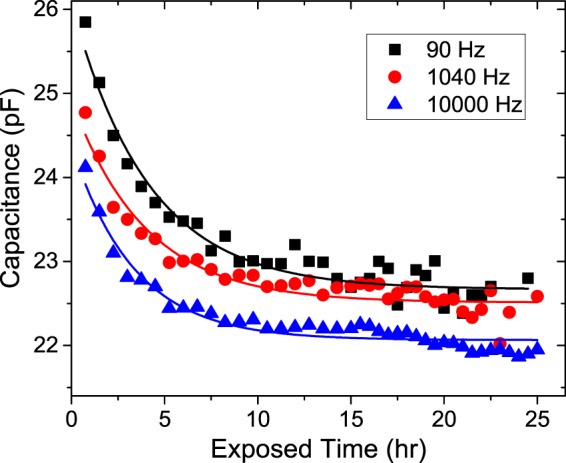
Capacitance decay measured at three different frequencies as a function of the exposure time during *in situ* hydrogen exposure at 10 MPa. The three solid lines are fitted with the equation C_0_ · exp(−A*D*_1_t) at the corresponding frequency.

The duration of exposure to H_2_ affected *C*_NBR_ (Fig. 5) at frequencies of 90, 1040, and 10,000 Hz during H_2_ exposure at a pressure of 10 MPa. Figure 5 is an enlargement of Fig. 4b and shows *C*_NBR_ as a function of exposure time at the three frequencies. *C*_NBR_ decreased exponentially with increasing exposure time to H_2_; this trend is caused by the diffusion of H_2_ into the NBR. In many polymers, such diffusion of high-pressure H_2_ induces plasticization^13–15^, which results in a reduction in the dielectric constant and capacitance. The decrease in the dielectric constant will be discussed later.

The change in the capacitance is expected only depend on the penetrated H_2_ content by assuming a first-order approximation. Thus, the evolution of the capacitance was affected by the diffusion-controlled process of H_2_. The content c(t) of H_2_ and the change in capacitance, which are the solutions of the diffusion equation, are expressed as follows^11^.

1 $$\begin{array}{c}{\rm{c}}({\rm{t}})=\frac{32}{{\pi }^{2}}c(0)\cdot [\mathop{\sum }\limits_{n=0}^{\infty }\,\frac{\exp (-{(2n+1)}^{2}{\pi }^{2}Dt/{z}^{2})}{{(2n+1)}^{2}}]\cdot [\mathop{\sum }\limits_{n=1}^{\infty }\,\frac{\exp (-\,D{\beta }_{n}^{2}t/{r}^{2})}{{\beta }_{n}^{2}}]\\ \approx \frac{32c(0)}{5.8{\pi }^{2}}\cdot exp[-(\frac{{\pi }^{2}}{{z}^{2}}+\frac{5.8}{{r}^{2}})Dt]\end{array}$$

where c(t) is the hydrogen content at time t and c(0) is the equilibrium hydrogen content. *D* is the diffusion coefficient (diffusivity) with a unit of [m^2^/s]. *z* and *r* are the thickness and radius of the disc-shaped specimen, respectively. *β*_*n*_ is the root of the zero-order Bessel function. We obtain the diffusion coefficient of H_2_ by fitting the data of the capacitance evolution to Eq. (1). The experimental data were well fitted to Eq. (1) by assuming a single-exponential decay of *C* = *C*_0_ ∙ exp(−A*D*_1_*t*) (Fig. 5, solid lines), where C_0_ is the capacitance at *t* = 0, and A is a constant corresponding to $(\frac{{\pi }^{2}}{{z}^{2}}+\frac{5.8}{{r}^{2}})$ with *z* = 2 mm and *r* = 24.5 mm. The obtained value of *D*_1_ is a diffusion coefficient that depends on *frequency* (Table 2) for the H_2_ penetration into the rubber.

**Table 2:** Diffusion coefficients *D*_1_ and *D*_2_ for pressurization (Fig. 5) and decompression (Fig. 6), respectively, at three frequencies.

Freq. (Hz)	*D*_1_(10^−11^ m^2^/s)	*D*_2_(10^−11^ m^2^/s)
90	3.1	2.4
1040	3.3	2.9
10000	3.4	3.1

**Figure 6:**
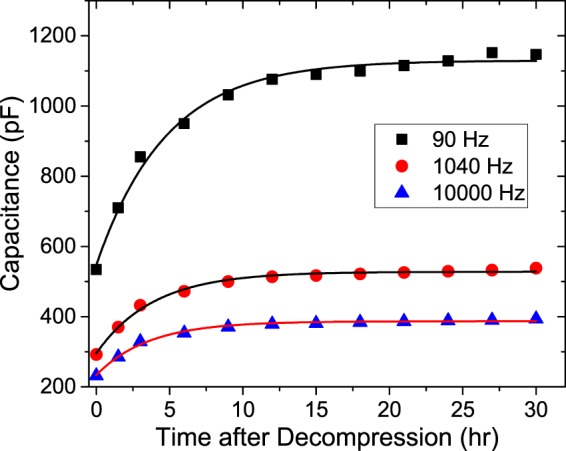
Capacitance recovery with time after decompression at a hydrogen pressure of 10 MPa. The three solid lines are the data fits to *C* = *C*_∞_ ∙ [1 − exp(−A*D*_2_*t*)] at each frequency.

Meanwhile, the recovery of the capacitance over time at atmospheric pressure after the decompression of the H_2_ pressure of 10 MPa exhibited an exponential increase at all frequencies to maxima that decreased with increasing *frequency* (Fig. 6). Figure 6 is an enlargement of Fig. 4c and presents *C*_NBR_ versus time after decompression. The exponential increase over time is a result of H_2_ desorption from the rubber by deplasticization after the release of pressure. The evolution of the capacitance was also controlled by the diffusion process of H_2_. An exponential function *C* = *C*_∞_ ∙ [1 − exp(−A*D*_2_*t*)], where C_∞_ is a saturated capacitance at infinite time, was also fitted to the data, and the diffusion coefficient *D*_2_ for the H_2_ desorption from the rubber was already obtained (Table 2). Single-exponential growth was also assumed for this fit.

The diffusion of H_2_ into the rubber saturates at ~12 h during pressurization (Fig. 5), and the clearance from the rubber during decompression takes ~15 h (Fig. 6). *D*_2_ is slightly larger than *D*_1_; this difference implies that the H_2_ desorption is slower than the absorption; therefore, a hysteretic phenomenon occurs, possibly because H_2_ interacts with the polymer chains.

We have also measured the penetrated H_2_ content into NBR as a function of time using TDA^11^ and electronic balances. According to the results in two methods, it takes about 10 h for the H_2_ contents to reach the maximum. This is consistent with the capacitance measurement as shown in Fig. 5. Thus, we could say the long-term change in capacitance is due to slow diffusion of H_2_. The results for decompression of Fig. 6 also shows similar behavior. The temperature and relative humidity were maintained within (23 ± 2) °C and (50 ± 5) %, respectively, in national calibration laboratory. The long-term drift effect on impedance analyser 4294A for several hours with a fresh specimen without H_2_ penetration was found to be negligible, which was less than approximately 3% of measured value. Therefore, the additional effects except of H_2_ was not included in the measurement.

**Figure 7:**
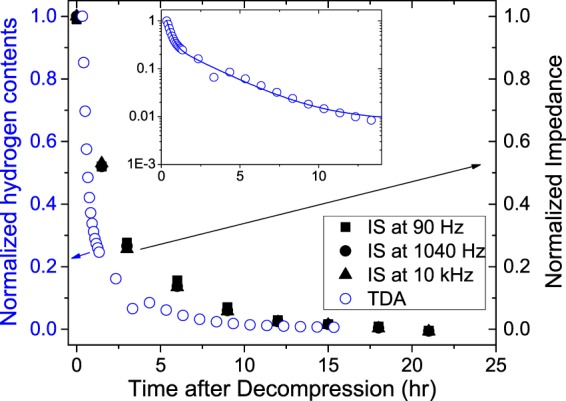
Comparison between the hydrogen content profile with time after decompression at a H_2_ pressure of 10 MPa measured by IS and TDA. Inset: exponential fitting (Solid Line) for the log-transformed data of the normalized hydrogen content.

*C*_NBR_ (Fig. 6) was transformed to the imaginary part of the impedance, *Z*_im_ = 1/(*ωC*) for a comparison with the TDA data. To find a correlation between IS and TDA, *Z*_im_ normalized by *C*_∞_ was compared with the normalized H_2_ content obtained by TDA (Fig. 7). The two results were consistent overall, which implies again that the change in *Z*_im_ is directly related to the desorbed H_2_ content. Such consistency is compatible with previous results obtained with TDA and NMR^16^. However, the IS data show a slower desorption of H_2_ than the TDA data (Fig. 7). Although there are unique measurement conditions with different dimensions in both of the specimens, this difference implies that the physics of the dependence of the impedance on the hydrogen content is different from that of the hydrogen diffusion process in the specimen. The TDA measurement only detects the hydrogen content released through the diffusion process. In addition to the effect of the released hydrogen content, IS is also affected by the chemical/physical interaction of hydrogen with the rubber polymer. Furthermore, the IS results may also due to the slow relaxation, i.e., the pressurization-induced state of the polymer matrix, or the fact that the filler distribution slowly relaxes towards another equilibrium and can continue to relax even after all the free H_2_ molecules are released during decompression. The relaxation may be irreversible; therefore, the original status cannot be recovered.

The TDA experimental data (Fig. 7) could not be fitted well with a single exponential function; therefore, a two-exponential fit on the basis of Eq. (1) was employed (Fig. 7, inset), exp(−B*D*_*2,f*_t) + exp(−B*D*_2,s_t), where B is a constant corresponding to $(\frac{{\pi }^{2}}{{z}^{2}}+\frac{5.8}{{r}^{2}})$ with *z* = 2 mm and *r* = 10 mm. *D*_2,f_ and *D*_2,s_ are fast and slow diffusion coefficients, respectively. The behaviour in the TDA data may arise from two kinds of diffusion processes for H_2_: a fast process (left side in the inset) for H_2_ in the polymer network matrix and a slow process (right side slope in the inset) from H_2_ in the filler (carbon black). *D*_2,f_ and *D*_2,s_ were found to be 3.9 × 10^−10^ m^2^/s and 4.2 × 10^−11^ m^2^/s, respectively.

**Figure 8:**
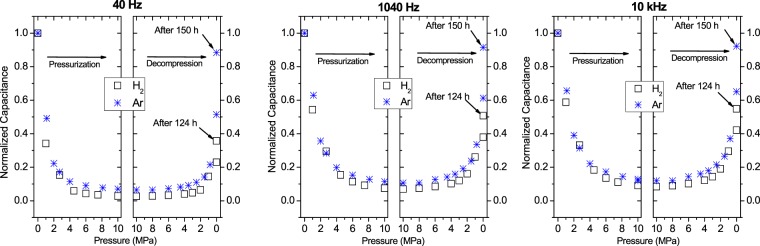
Change in the normalized capacitance with pressure in the pressurization and decompression processes under high-pressure hydrogen (ϒ) and argon (*) gases at three frequencies.

The normalized capacitance *C*_nor_ was also obtained during cyclic pressurization and decompression under H_2_ and Ar gases (Fig. 8). The pressure dependences of *C*_nor_ were similar at all three frequencies. *C*_nor_ decreased exponentially as the pressure increased during pressurization and later increased as the pressure decreased during decompression. The behaviours are very similar to those of *C*_NBR_ versus time (Figs 5 and 6).

The large change in the dielectric constant of the rubber at the low frequency of 40 Hz under high-pressure H_2_ (Fig. 8) can be explained by a change in the polarizability of the polymer chain matrix and the fillers, because the H_2_ in the rubber region contributes little to the total polarization. The strong frequency dependence of the dielectric constant of the rubber at the low frequency is explained by the polarization arising from the displacement or rotation of the fillers that have a net charge or a net dipole moment in the interface region around the fillers due to the interfacial polarization known as the Maxwell-Wagner-Sillars effect^17,18^.

The decrease in the dielectric constant with the exposure to high-pressure ambient H_2_ is expected, because the degrees of freedom of the polymer chain and the fillers decrease as the gas molecules occupy more space. A similar phenomenon was observed by pressurizing with Ar gas (Fig. 8).

According to the COMSOL simulation result *C*_NBR_ = 0.31 + 8.22*ε*_r_, the relative dielectric constant *ε*_r_ of the rubber sample at a pressure >5 MPa was found to be ~3, which has also been observed in silicon dioxide^19^ and many rigid plastic materials, such as epoxy glass^20^ and Bakelite^21^. A dielectric constant (~3) smaller than the value in a normal rubber polymer suggests that the decrease in the dielectric constant may occur because, due to the solvent, H_2_ causes the formation of a rigid plastic phase or a glass phase. This finding suggests that the penetration of high-pressure H_2_ with a low molecular mass in the process of pressurization, which generally induces plasticization for many polymers^13–15^, resulted in a reduction in the dielectric constant and capacitance of the polymers, whereas the increase in *C*_nor_ during decompression (Fig. 6) was caused by deplasticization after the release of pressure.

**Figure 9:**
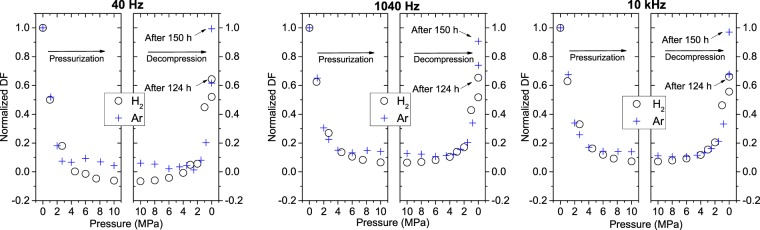
Change in the normalized DF with pressure in the pressurization and decompression processes under high-pressure hydrogen (○) and argon (+) gases at three frequencies.

The normalized *DF*_NBR_ was also affected by the pressure during both pressurization and decompression under both H_2_ and Ar gases at all three frequencies (Fig. 9). The behaviour was similar to that of *C*_nor_ under H_2_ and Ar gases. The measurement time for Figs 8 and 9 is about 2 h in the process of the pressurization and decompression. In Fig. 9, normalized dissipation factor (DF) value at 40 Hz is −0.06, for the capacitance of 25 pF in Fig. 8. In the case with the value of lowest frequency of 40 Hz and small capacitance under the applied AC voltage of 0.5 V, the magnitude of the measured DF is regarded as very small, because the 40 Hz is the lowest limit of the impedance analyzer 4294 A used and the manufacturer’s specification^22^ say the uncertainty of DF at this frequency and capacitance level amounts to 10%. Thus, the measured DF is virtually regarded as zero and the negative sign is attributed to the measuring instrument having large uncertainty of DF at low frequency of 40 Hz. However, with increasing the frequency, the uncertainty decreases.

The normalized *C*_NBR_ and normalized *DF*_NBR_ were affected more in H_2_ than in Ar during cyclic pressurization and decompression under the same pressure conditions (Figs 8 and 9). This difference implies that H_2_ has a greater permeation capability than Ar, possibly because of the molecular mass of H_2_. The most remarkable difference between H_2_ and Ar was that the recovery of the dielectric constant and the DF after decompression was considerably lower under H_2_ than under Ar (Figs 8 and 9). After 124 h of decompression in H_2_, the capacitance and the DF recovered to 40~60% of the initial values, whereas after 150 h of release in Ar, the rubber polymer recovered to 90~100% of the initial capacitance and DF. This failure to recover completely implies that H_2_ reacts chemically with the polymer and causes voids, defects, and a scission of the polymer chain, whereas inert Ar gas only causes a scission of the polymer. The SEM results partially support these findings, but further analysis is required.

### SEM results

**Figure 10:**
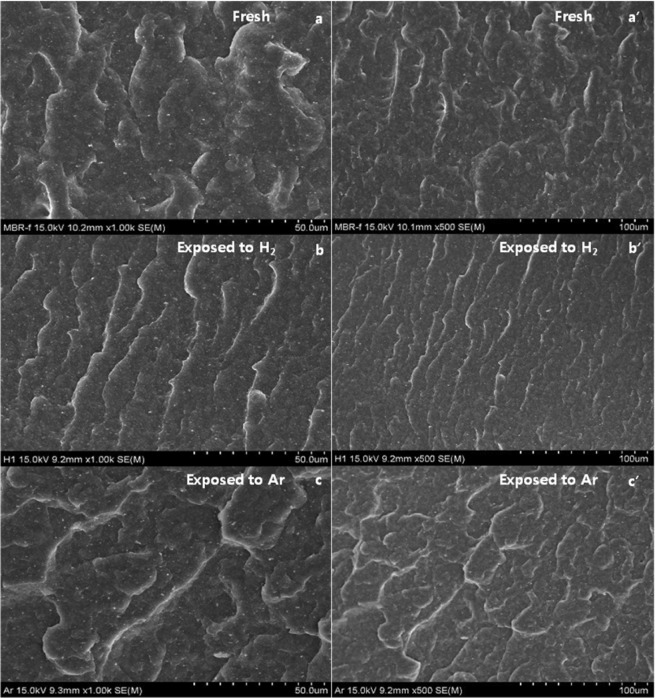
SEM images of the fresh NBR **(a,a’**) without exposure to hydrogen, NBR (**b,b’**) exposed to H_2_ at 10 MPa and NBR (**c,c’**) exposed to Ar at 10 MPa. Left column: image sequences obtained from the same spot; scale bar = 50 μm. Right column: sequences obtained from the same spot at a higher magnification; scale bar = 100 μm.

SEM images (Fig. 10) were obtained from the NBR without exposure to H_2_ and the NBR specimens exposed to H_2_ and Ar at 10 MPa. After exposure to H_2_, the morphology of the NBR (b, b’) changed from a random distribution to a uniaxial directed distribution. One possibility is that percolated channelling between low-density regions occurs during the permeation of H_2_ gas under 10 MPa of ambient pressure stress and causes a density modulation. In this instance, the distance between the valleys is ~10 μm. The NBR specimen exposed to Ar (c, c’) was modified to a circular type of modulation, which is reminiscent of a swollen balloon under gas injection instead of uniaxial channelling. The morphology of the Ar-exposed NBR reveals an Amoeba-like circle without any directional preference. These results demonstrate that different types of gases can result in different morphological responses at the same pressure. Thus we think it causes percolated channelling to an uniaxial directed distribution due to greater permeation than that exposed to Ar. Consequently, H_2_ effect on NBR are consistent with that already proposed by IS in views of greater permeation capability, stronger effects on physically and chemically in H_2_ more than Ar.

## Conclusions

This paper presents the development and evaluation of an IS system to measure the effects of high-pressure H_2_ gas on rubber polymers. The *in situ* capacitance measurement enables the observation of the correlation between macroscopic and microscopic phenomena under high pressure. The developed IS system could be used as an *in situ* probe to gather information such as diffusivity in H_2_ diffusion and desorption processes in rubber polymers.

Although there is a difference in the time evolutions between IS and TDA, the results of this study indicate that IS may be a useful probe to observe real-time and *in situ* changes in the H_2_ content with time and pressure during pressurization and subsequent decomposition and could supplement the *ex situ* TDA method and other methods. The proposed IS system could also clarify *in situ* the effect of the permeation properties of a gas as a function of time and pressure in rubber during sequential pressurization and decompression processes.

## References

[1] Züttel, A., Borgschulte, A. & Schlapbach, L. Hydrogen as a future energy carrier (ed. Züttel, A. *et al*.) 1–5 (Wiley-VCH Verlag GmbH, 2008).

[2] Ball M, Weeda M (2015). The hydrogen economy – vision or reality?. *Int. J. Hydrog*. Energy.

[3] Mazloomi K, Gomes C (2012). Hydrogen as an energy carrier: prospects and challenges. Renew. Sustain. Energy Rev..

[4] Marchi, C. W. & Somerday, B. P. Technical reference for hydrogen compatibility of materials. *Sandia National Laboratories*, http://www.sandia.gov/matlsTechRef (2008).

[5] Gangloff, R. & Somerday, B. Gaseous hydrogen embrittlement of materials in energy technologies: the problem, its characterisation and effects on particular alloy classes. (ed. Gangloff, R. *et al*.) 51–90 (Woodhead Publishing, 2012).

[6] Song J, Curtin WA (2013). Atomic mechanism and prediction of hydrogen embrittlement in iron. Nat. Mater..

[7] Louthan MR, Caskey GR, Donovan JA, Rawl DE (1972). Hydrogen embrittlement of metals. Mater. Sci. Eng..

[8] Murakami Y, Kanezaki T, Mine Y, Matsuoka S (2008). Hydrogen embrittlement mechanism in fatigue of austenitic stainless steels. Metall. Mater. Trans..

[9] Barth, R. R., Simmons, K. L. & Marchi, C. S. Polymers for hydrogen infrastructure and vehicle fuel systems: applications, properties, and gap analysis. Sandia National Laboratories, Sandia Report, Online ordering at, http://www.osti.gov/bridge (2013).

[10] Nishimura, S. International symposium of hydrogen polymers team, HYDROGENIUS, Kyushu University, http://hydrogenius.kyushu-u.ac.jp/ci/event/ihdf2017/pdf/prg-polver170111.pdf (2017).

[11] Yamabe J, Nishimura S (2009). Influence of fillers on hydrogen penetration properties and blister fracture of rubber composites for O-ring exposed to high-pressure hydrogen gas. Int. J. Hydrog. Energy.

[12] Yamabe J, Matsumoto T, Nishimura S (2011). Application of acoustic emission method to detection of internal fracture of sealing rubber material by high-pressure hydrogen decompression. Polym. Test..

[13] Chen X, Feng JJ, Bertelo CA (2006). Plasticization effects on bubble growth during polymer foaming. Polym. Eng. Sci..

[14] Bos A, Pünt IGM, Wessling M, Strathmann H (1999). CO_2_-induced plasticization phenomena in glassy *polymers*. J. Membr. Sci..

[15] Alessi P, Cortesi A, Kikic I, Vecchione F (2003). Plasticization of polymers with supercritical carbon dioxide: experimental determination of glass-transition temperatures. J. Appl. Polym. Sci..

[16] Nishimura S, Fujiwara H (2012). Detection of hydrogen dissolved in acrylonitrile butadiene rubber by ^1^H nuclear magnetic resonance. Chem. Phys. Lett..

[17] Mohanraj GT, Chaki TK, Chakraborty A, Khastgir D (2007). Measurement of AC conductivity and dielectric properties of flexible conductive styrene–butadiene rubber-carbon black composites. J. Appl. Polym. Sci..

[18] Samet M (2015). Electrode polarization vs. maxwell-wagner-sillars interfacial polarization in dielectric spectra of materials: characteristic frequencies and scaling laws. J. Chem. Phys..

[19] Gray, P. R., Hurst, P. J., Meyer, R. G. & Lewis, S. H. Analysis and design of analog integrated circuits. (ed. Vargas, V. A.) 40 (Wiley, 2009).

[20] Sokolov VI, Shalgunov SI, Gurtovnik IG, Mikheeva LG, Simonov-Emel’yanov ID (2005). Dielectric characteristics of glass fi bre reinforced plastics and their components. Int. Polym. Sci. Tech..

[21] IFM Relative dielectric constants, table of dielectric constants of substances, https://www.ifm.com/img/dialectric_constants.pdf, http://www.ydic.co.jp/english/technology/table_E.html.

[22] Agilent Technologies, Agilent 4294A Precision Impedance Analyzer Operation Manual Seventh Edition, Part No. 04294-90060, Chap. 10, 341–343 (2003).

